# Supplementary material for: Local decorin delivery via hyaluronic acid microrods improves cardiac performance, ventricular remodeling after myocardial infarction
Source: NPJ Regen Med. 2023 Oct 23;8:60. doi: 10.1038/s41536-023-00336-w (PMC10593781; doi:10.1038/s41536-023-00336-w)
Supplement: Supplementary file 1 — Supplementary Information [file 41536_2023_336_MOESM1_ESM.pdf]

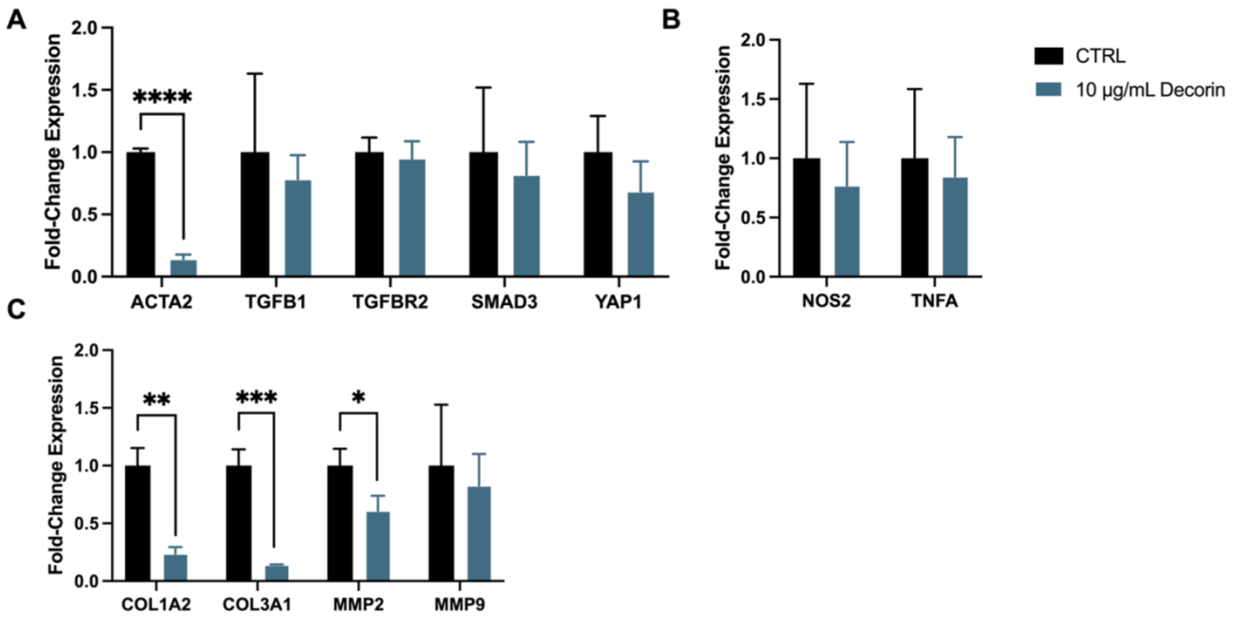

Supplemental Figure 1. Decorin decreases expression of fibrotic markers. Relative fold-change expression of genes relating to (A) TGF-β cascade, (B) inflammation, and (C) extracellular matrix in NIH 3T3 fibroblasts stimulated with either 10 ng/mL TGF-β1 (CTRL) or 10 ng/mL TGF-β1 plus 10 μg/mL decorin. Significant reduction in gene expression of ACTA2, COL1A2, COL3A1, and MMP2 were observed due to decorin treatment. The data are presented as the mean ± SD. *$p < 0.05$, **$p < 0.01$, ***$p < 0.001$, ****$p < 0.0001$.

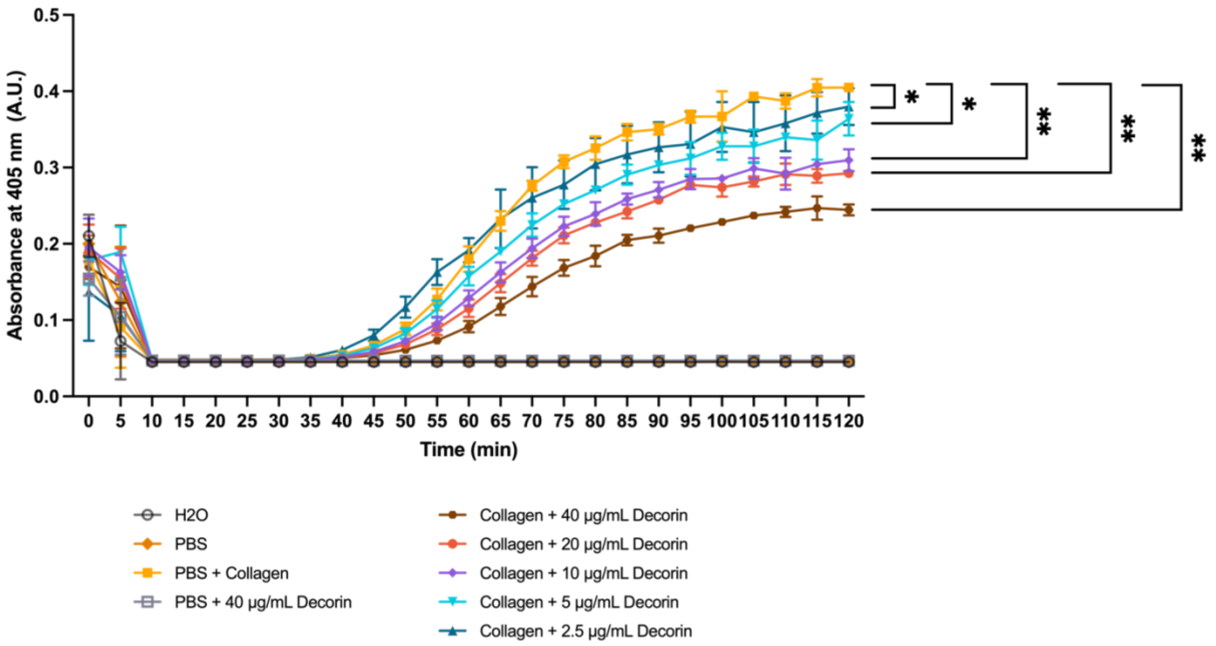

Supplemental Figure 2. Decorin decreases collagen fibril formation. Collagen fibrillization was decreased in a dose-dependent manner corresponding to the concentration of decorin administered. Only statistical significance of each decorin treated collagen group compared to PBS + Collagen control at the 120 min. timepoint is shown for clarity. The data are presented as the mean ± SD. *$p < 0.05$, **$p < 0.01$.

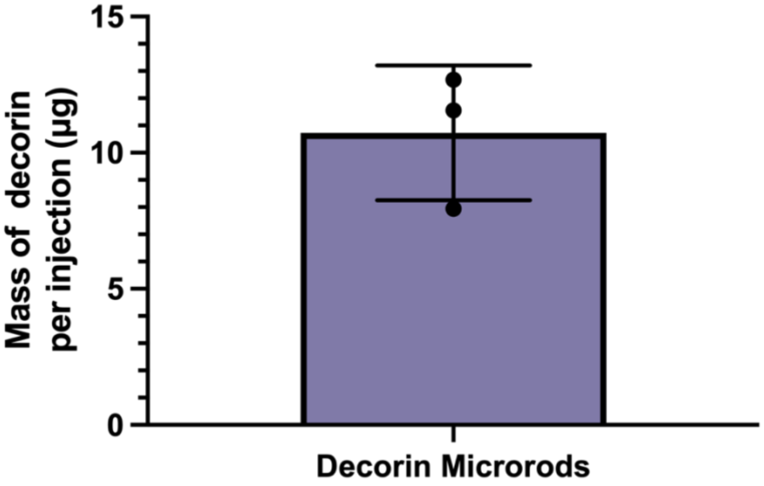

Supplemental Figure 3. Decorin loading per injection of 50,000 microrods. The amount of decorin loaded per injection (50,000 microrods) was calculated from three independent studies using protein quantitation. The average decorin loading was calculated to be approximately 10.7 µg of decorin per 50,000 microrods. The data are presented as the mean ± SD.

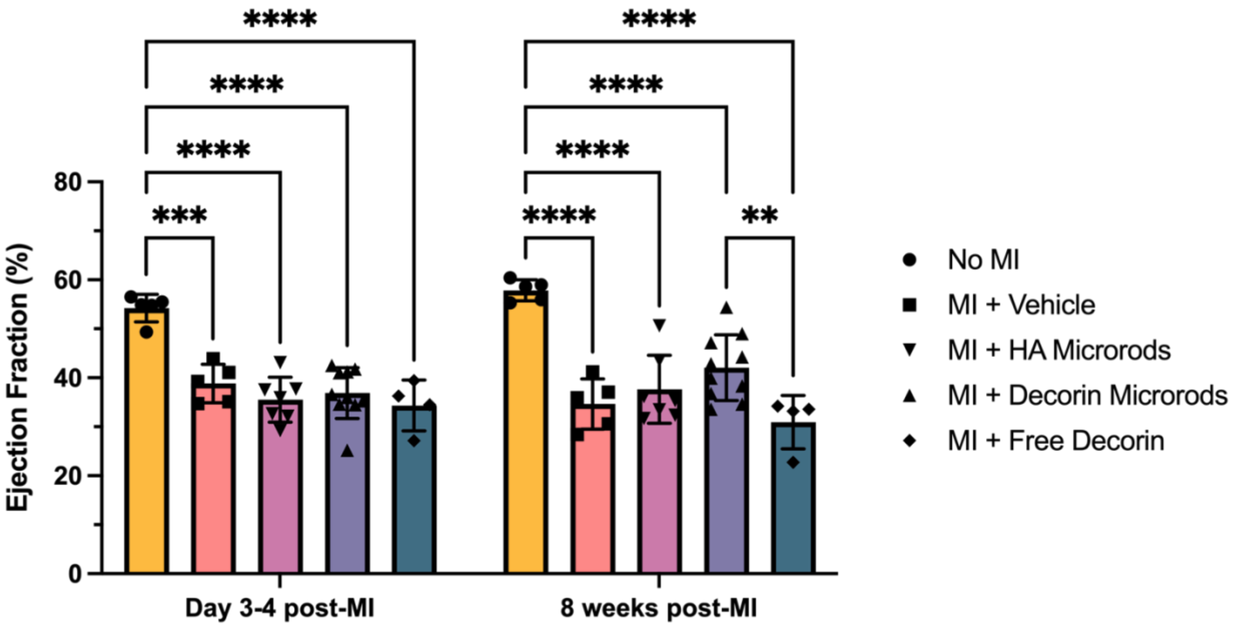

Supplemental Figure 4. Treatment with decorin microrods improves ejection fraction. Echocardiography was used to compare ejection fraction (EF) at 3-4 days after infarct and at 8 weeks after infarct in rats that had no MI performed (n = 5) and rats with MI that were treated with saline (n = 5), microrods (n = 7), decorin microrods (n = 10), and free decorin (n = 4). EF of all MI groups compared to No MI group are significantly decreased at Day 3–4, indicating a sufficient infarct. Rats treated with decorin microrods exhibited significantly higher EF compared to free decorin-treated animals after 8 weeks. The data are presented as the mean ± SD. **p < 0.01, ***p < 0.001, ****p < 0.0001.

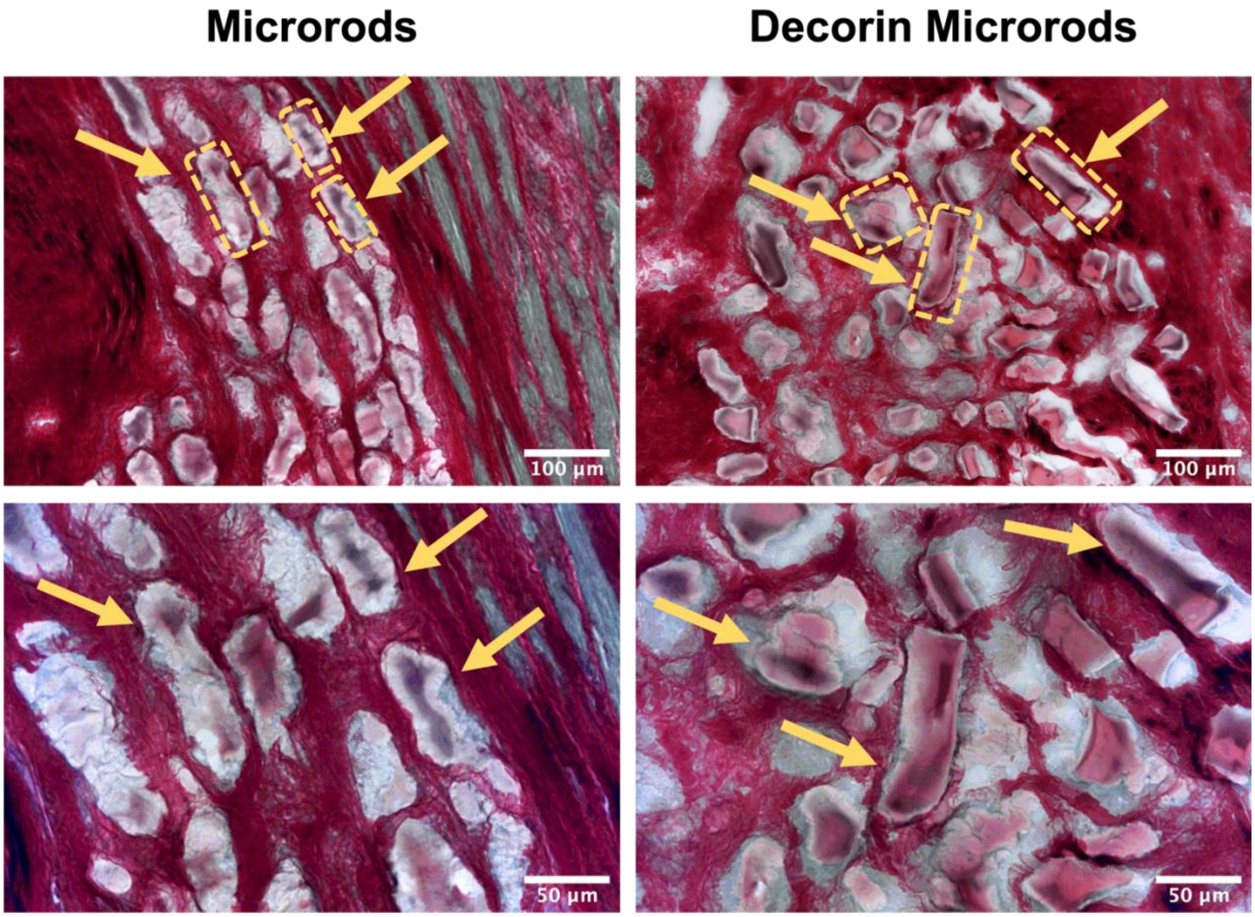

Supplemental Figure 5. Microrods remain in cardiac tissue for 8 weeks after injection. Microscopy revealed that microrods and decorin microrods (identified by arrows and outlined with dotted borders) were still present in cardiac tissue after 8 weeks. Scale bars = 100 µm (top) and 50 µm (bottom).

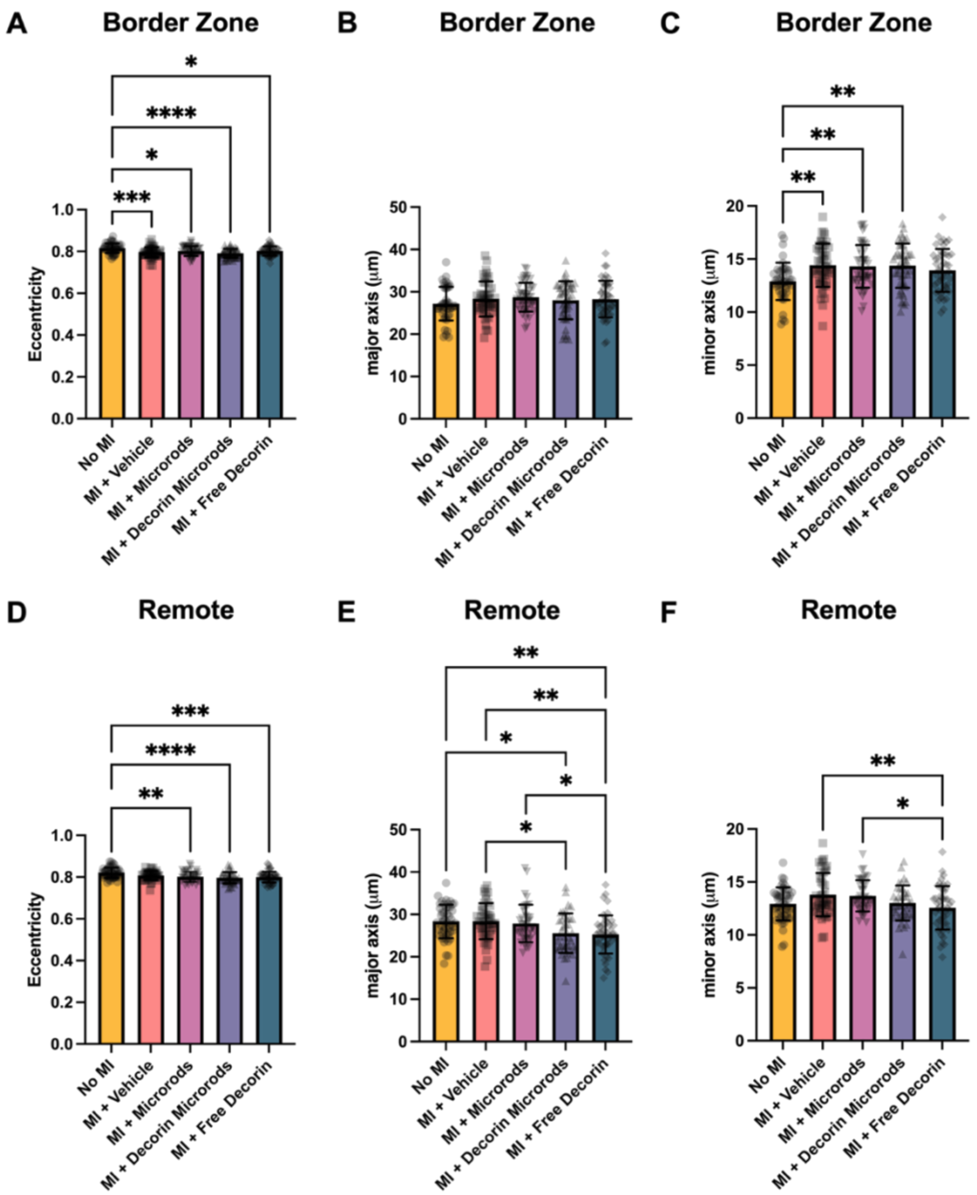

Supplemental Figure 6. Morphometric analysis of cardiomyocytes after treatment post-MI. Immunofluorescence staining for sarcomeric alpha actinin, cell membrane, and nuclei was performed to identify cardiomyocytes. Three tissue sections per heart (apex, middle, and end of the heart) were quantified for eccentricity, major axis length, and minor axis length were calculated for border zone (A-C, respectively) and remote zone (D-F, respectively) based on sarcomeric alpha actinin staining. In cases where there was not an identifiable remote zone for a given section in a particular animal, that section was excluded from data analysis for the remote zone of that animal. Analysis was performed by inverting images followed by binarization using an adaptive mean threshold before applying the watershed segmentation algorithm. While eccentricity was not identified to be different in either region between the experimental treatment groups, the remote zone in animals treated with decorin microrods and free decorin yielded smaller cells as assessed by major and minor axis lengths compared to those treated with saline and microrods. The data are presented as the mean ± SD. *$p < 0.05$, **$p < 0.01$, ***$p < 0.001$, ****$p < 0.0001$.

Supplemental Table 1. Primers used for qPCR.

Target Gene	Forward Primer (5’ -> 3’)	Reverse Primer (5’ -> 3’)
RPL19	CATTTTGCCCGACGAAAGGG	GATCTGCTGACGGGAGTTGG
ACTA2	GCTGCTCCAGCTATGTGTGA	CCATTCCAACCATTACTCCCTGA
COL1A2	AAGGGTGCTACTGGACTCCC	TTGTTACCGGATTCTCCTTTGG
COL3A1	CTGTAACATGGAAACTGGGGAAA	CCATAGCTGAACTGAAAACCACC
TGFB1	GGACTCTCCACCTGCAAGAC	CTGGCGAGCCTTAGTTTGGA
TGFBR2	ACGTTCCCAAGTCGGATGTG	TGTCGCAAGTGGACAGTCTC
SMAD3	AAGGCGACACATTGGGAGAG	GGGCAGCAAATTCCTGGTTG
MMP2	CAAGTTCCCCGGCGA TGTC	TTCTGGTCAAGGTCACCTGTC
MMP9	GGACCCGAAGCGGACATTG	CGTCGTCGAAATGGGCATCT
YAP1	TACTGATGCAGGTACTGCGG	TCAGGGATCTCAAAGGAGGAC
NOS2	GAGACAGGGAAGTCTGAAGCAC	CCAGCAGTAGTTGCTCCTCTTC
TNFA	TGGAACTGGCAGAAGAGG	AGACAGAAGAGCGTGGTG

Supplemental Table 2. *p* values for Figure 3A.

	Ejection Fraction		
**Šidák's multiple comparisons test**	**Predicted (LS) mean diff.**	**95.00% CI of diff.**	**Adjusted P Value**
Day 3-4 post-MI – 8 weeks post-MI			
No MI	-3.646	-8.073 to 0.7814	0.1456
MI + Vehicle	4.184	-0.2434 to 8.611	0.0707
MI + Microrods	-2.126	-5.868 to 1.616	0.4948
MI + Decorin Microrods	-5.212	-8.343 to -2.081	0.0005
MI + Free Decorin	3.420	-1.530 to 8.370	0.2919

Supplemental Table 3. *p* values for Figures 3B & 3E.

	Change in Ejection Fraction			Change in Stroke Volume		
**Tukey's multiple comparisons test**	**Mean Diff.**	**95.00% CI of diff.**	**Adjusted P Value**	**Mean Diff.**	**95.00% CI of diff.**	**Adjusted P Value**
No MI vs. MI + Vehicle	7.826	1.206 to 14.45	0.0147	23.63	-22.99 to 70.24	0.5813
No MI vs. MI + Microrods	1.520	-4.610 to 7.649	0.9485	-28.48	-71.64 to 14.68	0.3261
No MI vs. MI + Decorin Microrods	-1.568	-7.301 to 4.165	0.9280	-8.071	-48.44 to 32.30	0.9761
No MI vs. MI + Free Decorin	7.062	0.03954 to 14.08	0.0482	34.34	-15.11 to 83.78	0.2787
MI + Vehicle vs. MI + Microrods	-6.306	-12.44 to -0.1770	0.0415	-52.11	-95.27 to -8.947	0.0123
MI + Vehicle vs. MI + Decorin Microrods	-9.394	-15.13 to -3.661	0.0005	-31.70	-72.07 to 8.675	0.1772
MI + Vehicle vs. MI + Free Decorin	-0.7645	-7.786 to 6.257	0.9976	10.71	-38.73 to 60.16	0.9680
MI + Microrods vs. MI + Decorin Microrods	-3.088	-8.246 to 2.071	0.4211	20.41	-15.91 to 56.73	0.4834
MI + Microrods vs. MI + Free Decorin	5.542	-1.019 to 12.10	0.1279	62.82	16.62 to 109.0	0.0041
MI + Decorin Microrods vs. MI + Free Decorin	8.630	2.437 to 14.82	0.0032	42.41	-1.198 to 86.01	0.0595

Supplemental Table 4. *p* values for Supplemental Figure 4.

	Ejection Fraction		
**Tukey’s multiple comparisons test**	**Predicted (LS) mean diff.**	**95.00% CI of diff.**	**Adjusted P Value**
Day 3-4 post-MI			
No MI vs. MI + Vehicle	15.38	5.938 to 24.82	0.0003
No MI vs. MI + Microrods	18.74	9.996 to 27.48	<0.0001
No MI vs. MI + Decorin Microrods	17.33	9.151 to 25.51	<0.0001
No MI vs. MI + Free Decorin	19.87	9.854 to 29.88	<0.0001
MI + Vehicle vs. MI + Microrods	3.357	-5.384 to 12.10	0.8132
MI + Vehicle vs. MI + Decorin Microrods	1.948	-6.229 to 10.13	0.9613
MI + Vehicle vs. MI + Free Decorin	4.488	-5.526 to 14.50	0.7126
MI + Microrods vs. MI + Decorin Microrods	-1.409	-8.767 to 5.948	0.9825
MI + Microrods vs. MI + Free Decorin	1.131	-8.226 to 10.49	0.9970
MI + Decorin Microrods vs. MI + Free Decorin	2.541	-6.292 to 11.37	0.9255
8 weeks post-MI			
No MI vs. MI + Vehicle	23.21	13.77 to 32.65	<0.0001
No MI vs. MI + Microrods	20.26	11.52 to 29.00	<0.0001
No MI vs. MI + Decorin Microrods	15.76	7.585 to 23.94	<0.0001
No MI vs. MI + Free Decorin	26.93	16.92 to 36.95	<0.0001
MI + Vehicle vs. MI + Microrods	-2.952	-11.69 to 5.789	0.8740
MI + Vehicle vs. MI + Decorin Microrods	-7.448	-15.63 to 0.7291	0.0903
MI + Vehicle vs. MI + Free Decorin	3.725	-6.290 to 13.74	0.8303
MI + Microrods vs. MI + Decorin Microrods	-4.496	-11.85 to 2.862	0.4270
MI + Microrods vs. MI + Free Decorin	6.677	-2.681 to 16.03	0.2728
MI + Decorin Microrods vs. MI + Free Decorin	11.17	2.340 to 20.00	0.0066

Supplemental Table 5. *p* values for Figures 3C-D.

	End Systolic Volume			End Diastolic Volume		
**Tukey's multiple comparisons test**	**Predicted (LS) mean diff.**	**95.00% CI of diff.**	**Adjusted P Value**	**Predicted (LS) mean diff.**	**95.00% CI of diff.**	**Adjusted P Value**
Day 3-4 post-MI						
No MI vs. MI + Vehicle	-108.4	-285.8 to 68.99	0.4270	-53.40	-245.8 to 139.0	0.9341
No MI vs. MI + Microrods	-132.3	-296.6 to 31.89	0.1688	-61.60	-239.7 to 116.5	0.8643
No MI vs. MI + Decorin Microrods	-88.50	-242.1 to 65.13	0.4869	-3.600	-170.2 to 163.0	>0.9999
No MI vs. MI + Free Decorin	-181.2	-369.4 to 6.953	0.0643	-127.6	-331.7 to 76.48	0.4036
MI + Vehicle vs. MI + Microrods	-23.94	-188.2 to 140.3	0.9938	-8.200	-186.3 to 169.9	>0.9999
MI + Vehicle vs. MI + Decorin Microrods	19.90	-133.7 to 173.5	0.9960	49.80	-116.8 to 216.4	0.9153
MI + Vehicle vs. MI + Free Decorin	-72.80	-261.0 to 115.4	0.8091	-74.20	-278.3 to 129.9	0.8416
MI + Microrods vs. MI + Decorin Microrods	43.84	-94.38 to 182.1	0.8970	58.00	-91.93 to 207.9	0.8092
MI + Microrods vs. MI + Free Decorin	-48.86	-224.7 to 126.9	0.9337	-66.00	-256.7 to 124.7	0.8639
MI + Decorin Microrods vs. MI + Free Decorin	-92.70	-258.6 to 73.24	0.5175	-124.0	-304.0 to 55.99	0.3063
8 weeks post-MI						
No MI vs. MI + Vehicle	-331.4	-508.8 to -154.0	<0.0001	-252.8	-445.2 to -60.39	0.0044
No MI vs. MI + Microrods	-323.0	-487.2 to -158.7	<0.0001	-280.5	-458.6 to -102.3	0.0004
No MI vs. MI + Decorin Microrods	-163.1	-316.7 to -9.474	0.0323	-86.40	-253.0 to 80.23	0.5892
No MI vs. MI + Free Decorin	-416.7	-604.8 to -228.5	<0.0001	-328.9	-532.9 to -124.8	0.0003
MI + Vehicle vs. MI + Microrods	8.429	-155.8 to 172.7	0.9999	-27.66	-205.8 to 150.5	0.9921
MI + Vehicle vs. MI + Decorin Microrods	168.3	14.67 to 321.9	0.0251	166.4	-0.2341 to 333.0	0.0505
MI + Vehicle vs. MI + Free Decorin	-85.25	-273.4 to 102.9	0.7043	-76.05	-280.1 to 128.0	0.8293
MI + Microrods vs. MI + Decorin Microrods	159.9	21.65 to 298.1	0.0158	194.1	44.13 to 344.0	0.0052
MI + Microrods vs. MI + Free Decorin	-93.68	-269.5 to 82.12	0.5635	-48.39	-239.1 to 142.3	0.9516
MI + Decorin Microrods vs. MI + Free Decorin	-253.6	-419.5 to -87.61	0.0007	-242.5	-422.4 to -62.46	0.0033

Supplemental Table 6. *p* values for Figures 4B-C.

	Average Wall Thickness			Average Intensity in LV		
**Tukey's multiple comparisons test**	**Mean Diff.**	**95.00% CI of diff.**	**Adjusted P Value**	**Mean Diff.**	**95.00% CI of diff.**	**Adjusted P Value**
No MI vs. MI + Vehicle	1163	375.2 to 1952	0.0017	-769205759	-1446352225 to -92059293	0.0202
No MI vs. MI + Microrods	1014	284.5 to 1744	0.0033	-91091658	-718007467 to 535824151	0.9927
No MI vs. MI + Decorin Microrods	798.5	116.0 to 1481	0.0160	-79066514	-665492556 to 507359528	0.9945
No MI vs. MI + Free Decorin	1396	559.7 to 2232	0.0004	-93589275	-811811563 to 624633012	0.9952
MI + Vehicle vs. MI + Microrods	-149.1	-878.8 to 580.6	0.9741	678114101	51198292 to 1305029910	0.0293
MI + Vehicle vs. MI + Decorin Microrods	-364.8	-1047 to 317.8	0.5317	690139245	103713203 to 1276565287	0.0152
MI + Vehicle vs. MI + Free Decorin	232.3	-603.7 to 1068	0.9240	675616484	-42605804 to 1393838771	0.0726
MI + Microrods vs. MI + Decorin Microrods	-215.7	-829.8 to 398.4	0.8399	12025144	-515603132 to 539653420	>0.9999
MI + Microrods vs. MI + Free Decorin	381.4	-399.7 to 1163	0.6147	-2497617	-673570899 to 668575664	>0.9999
MI + Decorin Microrods vs. MI + Free Decorin	597.1	-140.2 to 1334	0.1552	-14522761	-647935281 to 618889758	>0.9999

Supplemental Table 7. *p* values for Figures 5B-C.

	Border Zone (Area)			Remote (Area)		
**Tukey's multiple comparisons test**	**Mean Diff.**	**95.00% CI of diff.**	**Adjusted P Value**	**Mean Diff.**	**95.00% CI of diff.**	**Adjusted P Value**
No MI vs. MI + Vehicle	-61.16	-113.6 to -8.718	0.0132	-31.33	-78.48 to 15.82	0.3601
No MI vs. MI + Microrods	-67.17	-119.6 to -14.72	0.0047	-16.00	-64.36 to 32.37	0.8928
No MI vs. MI + Decorin Microrods	-58.79	-111.2 to -6.348	0.0194	19.44	-28.92 to 67.81	0.8033
No MI vs. MI + Free Decorin	-49.47	-101.9 to 2.981	0.0749	29.27	-16.84 to 75.38	0.4080
MI + Vehicle vs. MI + Microrods	-6.002	-57.29 to 45.29	0.9977	15.33	-34.02 to 64.69	0.9129
MI + Vehicle vs. MI + Decorin Microrods	2.370	-48.92 to 53.66	>0.9999	50.78	1.421 to 100.1	0.0403
MI + Vehicle vs. MI + Free Decorin	11.70	-39.59 to 62.99	0.9706	60.60	13.45 to 107.8	0.0045
MI + Microrods vs. MI + Decorin Microrods	8.371	-42.92 to 59.66	0.9916	35.44	-15.07 to 85.96	0.3046
MI + Microrods vs. MI + Free Decorin	17.70	-33.59 to 68.99	0.8774	45.27	-3.096 to 93.63	0.0787
MI + Decorin Microrods vs. MI + Free Decorin	9.329	-41.96 to 60.62	0.9873	9.827	-38.54 to 58.19	0.9807

Supplemental Table 8. *p* values for Figures 5D-E.

	Border Zone (Cells per Area)			Remote (Cells per Area)		
**Tukey's multiple comparisons test**	**Mean Diff.**	**95.00% CI of diff.**	**Adjusted P Value**	**Mean Diff.**	**95.00% CI of diff.**	**Adjusted P Value**
No MI vs. MI + Vehicle	0.0004279	0.0001550 to 0.0007009	0.0002	0.0002227	-7.803e-005 to 0.0005235	0.2518
No MI vs. MI + Microrods	0.0005185	0.0002455 to 0.0007914	<0.0001	4.902e-005	-0.0002595 to 0.0003575	0.9924
No MI vs. MI + Decorin Microrods	0.0004200	0.0001470 to 0.0006929	0.0003	-0.0001497	-0.0004582 to 0.0001588	0.6697
No MI vs. MI + Free Decorin	0.0004351	0.0001622 to 0.0007081	0.0002	-0.0001204	-0.0004146 to 0.0001737	0.7925
MI + Vehicle vs. MI + Microrods	9.053e-005	-0.0001764 to 0.0003575	0.8840	-0.0001737	-0.0004885 to 0.0001411	0.5520
MI + Vehicle vs. MI + Decorin Microrods	-7.958e-006	-0.0002749 to 0.0002590	>0.9999	-0.0003724	-0.0006872 to -5.762e-005	0.0114
MI + Vehicle vs. MI + Free Decorin	7.180e-006	-0.0002598 to 0.0002741	>0.9999	-0.0003431	-0.0006439 to -4.239e-005	0.0164
MI + Microrods vs. MI + Decorin Microrods	-9.849e-005	-0.0003654 to 0.0001684	0.8486	-0.0001987	-0.0005209 to 0.0001235	0.4381
MI + Microrods vs. MI + Free Decorin	-8.335e-005	-0.0003503 to 0.0001836	0.9116	-0.0001694	-0.0004779 to 0.0001391	0.5565
MI + Decorin Microrods vs. MI + Free Decorin	1.514e-005	-0.0002518 to 0.0002821	0.9999	2.929e-005	-0.0002792 to 0.0003378	0.9990

Supplemental Table 9. *p* values for Figure 6.

	Infarct (# Vessels/Field)			Border Zone (# Vessels/Field)			Remote (# Vessels/Field)		
**Tukey's multiple comparisons test**	**Mean Diff.**	**95.00% CI of diff.**	**Adjusted P Value**	**Mean Diff.**	**95.00% CI of diff.**	**Adjusted P Value**	**Mean Diff.**	**95.00% CI of diff.**	**Adjusted P Value**
MI + Vehicle vs. MI + Microrods	-0.2708	-0.8198 to 0.2781	0.5776	-0.2292	-0.7146 to 0.2563	0.6126	-0.1750	-0.7107 to 0.3607	0.8312
MI + Vehicle vs. MI + Decorin Microrods	-0.2500	-0.7990 to 0.2990	0.6399	-0.1250	-0.6104 to 0.3604	0.9092	-0.4500	-0.9857 to 0.08573	0.1330
MI + Vehicle vs. MI + Free Decorin	0.2708	-0.2781 to 0.8198	0.5776	-0.1250	-0.6104 to 0.3604	0.9092	0.4000	-0.1357 to 0.9357	0.2161
MI + Microrods vs. MI + Decorin Microrods	0.02083	-0.5281 to 0.5698	0.9997	0.1042	-0.3813 to 0.5896	0.9448	-0.2750	-0.8107 to 0.2607	0.5433
MI + Microrods vs. MI + Free Decorin	0.5417	-0.007312 to 1.091	0.0546	0.1042	-0.3813 to 0.5896	0.9448	0.5750	0.03927 to 1.111	0.0301
MI + Decorin Microrods vs. MI + Free Decorin	0.5208	-0.02815 to 1.070	0.0698	0.000	-0.4854 to 0.4854	>0.9999	0.8500	0.3143 to 1.386	0.0004
