# Supplementary material for: Local decorin delivery via hyaluronic acid microrods improves cardiac performance, ventricular remodeling after myocardial infarction
Source: NPJ Regen Med. 2023 Oct 23;8:60. doi: 10.1038/s41536-023-00336-w (PMC10593781; doi:10.1038/s41536-023-00336-w)
Supplement: Supplementary file 2 — Reporting Summary [file 41536_2023_336_MOESM2_ESM.pdf]

nature portfolio

Corresponding author(s): Tejal A. Desai

Last updated by author(s): Oct 4, 2023

Reporting Summary

Nature Portfolio wishes to improve the reproducibility of the work that we publish. This form provides structure for consistency and transparency in reporting. For further information on Nature Portfolio policies, see our Editorial Policies and the Editorial Policy Checklist.

Statistics

For all statistical analyses, confirm that the following items are present in the figure legend, table legend, main text, or Methods section.

n/a	Confirmed	
☐	☒	The exact sample size (*n*) for each experimental group/condition, given as a discrete number and unit of measurement
☐	☒	A statement on whether measurements were taken from distinct samples or whether the same sample was measured repeatedly
☐	☒	The statistical test(s) used AND whether they are one- or two-sided<br>*Only common tests should be described solely by name; describe more complex techniques in the Methods section.*
☒	☐	A description of all covariates tested
☐	☒	A description of any assumptions or corrections, such as tests of normality and adjustment for multiple comparisons
☐	☒	A full description of the statistical parameters including central tendency (e.g. means) or other basic estimates (e.g. regression coefficient) AND variation (e.g. standard deviation) or associated estimates of uncertainty (e.g. confidence intervals)
☒	☐	For null hypothesis testing, the test statistic (e.g. *F*, *t*, *r*) with confidence intervals, effect sizes, degrees of freedom and *P* value noted<br>*Give P values as exact values whenever suitable.*
☒	☐	For Bayesian analysis, information on the choice of priors and Markov chain Monte Carlo settings
☒	☐	For hierarchical and complex designs, identification of the appropriate level for tests and full reporting of outcomes
☒	☐	Estimates of effect sizes (e.g. Cohen's *d*, Pearson's *r*), indicating how they were calculated

Our web collection on statistics for biologists contains articles on many of the points above.

Software and code

Policy information about availability of computer code

Data collection	Nikon Elements AR 5.21.03, build 1489 was used to collect brightfield and immunofluorescence images; Quantstudio Realtime PCR v1.6.1 was used to collect gene expression data, SpectraMax Pro 7 was used to collect absorbance data
Data analysis	ImageJ 1.53 was used to analyze histology and immunofluorescence (vascularization) images, custom code used to analyze hypertrophy images will be made available upon request.

For manuscripts utilizing custom algorithms or software that are central to the research but not yet described in published literature, software must be made available to editors and reviewers. We strongly encourage code deposition in a community repository (e.g. GitHub). See the Nature Portfolio guidelines for submitting code & software for further information.

Data

Policy information about availability of data

All manuscripts must include a data availability statement. This statement should provide the following information, where applicable:

- Accession codes, unique identifiers, or web links for publicly available datasets
- A description of any restrictions on data availability
- For clinical datasets or third party data, please ensure that the statement adheres to our policy

Raw data that support the graphs within this paper are available from the authors upon reasonable request.

Research involving human participants, their data, or biological material

Policy information about studies with human participants or human data. See also policy information about sex, gender (identity/presentation), and sexual orientation and race, ethnicity and racism.

Reporting on sex and gender	This study did not involve human participants, their data, or biological material
Reporting on race, ethnicity, or other socially relevant groupings	This study did not involve human participants, their data, or biological material
Population characteristics	This study did not involve human participants, their data, or biological material
Recruitment	This study did not involve human participants, their data, or biological material
Ethics oversight	This study did not involve human participants, their data, or biological material

Note that full information on the approval of the study protocol must also be provided in the manuscript.

Field-specific reporting

Please select the one below that is the best fit for your research. If you are not sure, read the appropriate sections before making your selection.

☒ Life sciences ☐ Behavioural & social sciences ☐ Ecological, evolutionary & environmental sciences

For a reference copy of the document with all sections, see nature.com/documents/nr-reporting-summary-flat.pdf

Life sciences study design

All studies must disclose on these points even when the disclosure is negative.

Sample size	No statistical methods were used to predetermine the same size. Sample sizes were estimated based on previous experiences and reports with similar setups that showed statistical significance, for example reference 13. Subsequent power analysis using G*power showed sufficient power with the current sample size treatment group numbers (a=0.05, total sample size=31, # groups=5, effect size f=1.23)
Data exclusions	Animals whose baseline ejection fraction (Day 3-4 post-MI) was above 45% were excluded because they indicated an insufficient infarct model. In certain situations, when three sections (apex, middle, and end of the heart) for infarct, border zone, and remote zone were analyzed for hypertrophy and vascular analysis, if sections did not have a distinguishable "remote" region, these slices were excluded from the analysis.
Replication	The experimental repeats for individual assays are specified in the methods and/or figure legend.
Randomization	At Days 3-4 post-MI, animals were imaged via echocardiography and categorized into three separate bins "mild infarct", "medium infarct", and "severe infarct". Animals categorized as mild, medium, and severe infarct were then randomly distributed to saline, microrod, decorin microrod, or free decorin groups so as to allocate approximately equivalent numbers of each infarct severity to each treatment group.
Blinding	The investigator who was performing the treatment injections was blinded as to which treatment was being injected into each animal and animals were assigned a random ID tag. Individuals who analyzed the echocardiography, hypertrophy, and vascularization images were only informed of the animal ID and had no knowledge of the treatment condition.

Reporting for specific materials, systems and methods

We require information from authors about some types of materials, experimental systems and methods used in many studies. Here, indicate whether each material, system or method listed is relevant to your study. If you are not sure if a list item applies to your research, read the appropriate section before selecting a response.

Materials & experimental systems

n/a	Involved in the study
☐	☒ Antibodies
☐	☒ Eukaryotic cell lines
☒	☐ Palaeontology and archaeology
☐	☒ Animals and other organisms
☒	☐ Clinical data
☒	☐ Dual use research of concern
☒	☐ Plants

Methods

n/a	Involved in the study
☒	☐ ChIP-seq
☒	☐ Flow cytometry
☒	☐ MRI-based neuroimaging

Antibodies

Antibodies used	Anti-decorin antibody (ab151988) - polyclonal, anti-sarcomeric alpha actinin (ab137346) - polyclonal, and anti-alpha smooth muscle actin (ab5694) - polyclonal were purchased from Abcam
Validation	Anti-decorin antibody (ab151988) is quality controlled by the vendor and is cited by 4 peer reviewed papers (https://www.abcam.com/products/primary-antibodies/decorin-antibody-ab151988.html)<br>Anti-sarcomeric alpha actinin (ab137346) is quality controlled by the vendor and is cited by 35 peer reviewed papers (https://www.abcam.com/products/primary-antibodies/sarcomeric-alpha-actinin-antibody-ab137346.html)<br>Anti-alpha smooth muscle actin (ab5694) is quality controlled by the vendor and is cited by 2188 peer reviewed papers (https://www.abcam.com/products/primary-antibodies/alpha-smooth-muscle-actin-antibody-ab5694.html)

Eukaryotic cell lines

Policy information about cell lines and Sex and Gender in Research

Cell line source(s)	NIH 3T3 (ATCC)
Authentication	The cells were purchased from the vendor ATCC and so validation was not performed.
Mycoplasma contamination	This cell line was not tested for mycoplasma contamination after purchase from the vendor.
Commonly misidentified lines (See ICLAC register)	No commonly misidentified cell lines were used.

Animals and other research organisms

Policy information about studies involving animals; ARRIVE guidelines recommended for reporting animal research, and Sex and Gender in Research

Laboratory animals	Male Sprague-Dawley rats (200–225g)
Wild animals	No wild animals were used
Reporting on sex	Findings only apply to the male sex. It has been documented that there may exist differences in post-MI left ventricular remodeling based that is influenced by sex. Prior studies have demonstrated favorable remodeling processes in females compared to males after myocardial injury that may be due to the ability to retain advantageous myocardial properties, such as reduced myocyte apoptosis and hypertrophy (references 47–51).
Field-collected samples	This study did not involve samples collected from the field.
Ethics oversight	Committee for Animal Research of the University of California, San Francisco

Note that full information on the approval of the study protocol must also be provided in the manuscript.
